# 655. Enhancing Staff Knowledge of Sleep Hygiene for Burn Patient Recovery

**DOI:** 10.1093/jbcr/irag033.492

**Published:** 2026-04-06

**Authors:** Lisa C Smith, Sheera F Lerman, Carrie A Cox, Shobha Ambi, Yvette Jenifer, Emily H Werthman, Kathryn Reopel, Sherry Campbell-Heim, Cristian Cazac, Julie A Caffrey

**Affiliations:** Johns Hopkins Bayview Medical Center, Baltimore, MD; Johns Hopkins University School of Medicine, Baltimore, MD; Johns Hopkins Burn Center, Baltimore, MD; Johns Hopkins University School of Nursing, Baltimore, MD; Johns Hopkins Medicine, Baltimore, MD; Johns Hopkins Burn Center, Baltimore, MD; Johns Hopkins Bayview Medical Center, Baltimore, MD; Johns Hopkins Bayview Burn ICU, Baltimore, MD; Johns Hopkins University School of Medicine, Baltimore, MD; Johns Hopkins University School of Medicine, Baltimore, MD

## Abstract

**Introduction:**

Burn patients often experience sleep disturbances during their recovery including difficulties falling asleep, frequent awakenings, early morning waking, and poor sleep quality. Common causes of these sleep problems include pain, PTSD, and depression. While hospital staff generally understand the importance of sleep, this quality improvement project explores whether a structured intervention can enhance staff awareness and implementation of sleep hygiene practices to support recovery in burn patients.

**Methods:**

An anonymous survey of nurses and patient care technicians evaluated their knowledge of sleep hygiene and perceptions of patients’ sleep quality. An educational program on sleep disturbances was delivered, accompanied by a sleep hygiene protocol incorporating daytime and nighttime intervention bundles. Staff were trained on sleep assessment documentation, and the protocol was posted in patients’ rooms as well as on education boards throughout the burn center. Sleep hygiene champions were designated on both shifts to address questions and support adherence.

**Results:**

Prior to implementation, 96% of staff recognized the health risks associated with sleep deprivation, and 89% understood that frequent daytime naps negatively affect nighttime sleep quality. Additionally, 60% reported that patients typically awaken independently three or more times a night and 69% noted that patients were awakened three or more times for nightly interventions. Documentation of daily patient sleep assessments increased from 24% to 31% following the protocol launch.

The protocol’s launch was delayed until mid-August 2025 due to challenges in ensuring patients receive 7-8 hours of minimally interrupted sleep. Barriers included high staff turnover and unfamiliarity with the sleep hygiene protocol among visiting staff and off-service patients’ care teams. Coordination of non-urgent care interventions and medication timing also posed challenges. Post-implementation surveys are planned to follow a three-month interval to evaluate progress and guide further improvements.

**Conclusions:**

Nursing staff have a good understanding of the importance of sleep, however there are significant barriers to implementing sleep hygiene protocols in the inpatient burn center setting. The new sleep hygiene protocol was designed to address some of these barriers and a post implementation survey will be administered three months after implementation to assess changes in knowledge and protocol adherence.

**Applicability of Research to Practice:**

Improving staff knowledge and practicing sleep hygiene is crucial in optimizing recovery for burn patients. Structured protocols, combined with education and reinforcement, can promote sleep-friendly care environments in burn units.

**Funding for the study:**

N/A.

